# Efficient Detection of Pathogenic Leptospires Using 16S Ribosomal RNA

**DOI:** 10.1371/journal.pone.0128913

**Published:** 2015-06-19

**Authors:** Brian T. Backstedt, Ozlem Buyuktanir, Janet Lindow, Elsio A. Wunder, Mitermayer G. Reis, Sahar Usmani-Brown, Michel Ledizet, Albert Ko, Utpal Pal

**Affiliations:** 1 Department of Veterinary Medicine and Virginia-Maryland Regional College of Veterinary Medicine, University of Maryland, College Park, Maryland, United States of America; 2 Department of Epidemiology of Microbial Diseases, Yale University School of Public Health, New Haven, Connecticut, United States of America; 3 Centro de Pesquisas Gonçalo Moniz, Fundação Oswaldo Cruz, Salvador, Brazil; 4 L2 Diagnostics, New Haven, Connecticut, United States of America; University of Kentucky College of Medicine, UNITED STATES

## Abstract

Pathogenic *Leptospira* species cause a prevalent yet neglected zoonotic disease with mild to life-threatening complications in a variety of susceptible animals and humans. Diagnosis of leptospirosis, which primarily relies on antiquated serotyping methods, is particularly challenging due to presentation of non-specific symptoms shared by other febrile illnesses, often leading to misdiagnosis. Initiation of antimicrobial therapy during early infection to prevent more serious complications of disseminated infection is often not performed because of a lack of efficient diagnostic tests. Here we report that specific regions of leptospiral 16S ribosomal RNA molecules constitute a novel and efficient diagnostic target for PCR-based detection of pathogenic *Leptospira* serovars. Our diagnostic test using spiked human blood was at least 100-fold more sensitive than corresponding leptospiral DNA-based quantitative PCR assays, targeting the same 16S nucleotide sequence in the RNA and DNA molecules. The sensitivity and specificity of our RNA assay against laboratory-confirmed human leptospirosis clinical samples were 64% and 100%, respectively, which was superior then an established parallel DNA detection assay. Remarkably, we discovered that 16S transcripts remain appreciably stable *ex vivo*, including untreated and stored human blood samples, further highlighting their use for clinical detection of *L*. *interrogans*. Together, these studies underscore a novel utility of RNA targets, specifically 16S rRNA, for development of PCR-based modalities for diagnosis of human leptospirosis, and also may serve as paradigm for detection of additional bacterial pathogens for which early diagnosis is warranted.

## Introduction

Leptospirosis is a prominent zoonotic disease caused by a diverse group of pathogenic leptospires that includes at least nine genospecies and over 200 serovars [1–7]. There are an estimated more than 800,000 cases and 50,000 deaths annually due to leptospirosis [8]. The greatest disease burden occurs in subsistence farmers [9, 10] and urban slum dwellers [2, 11–13], especially in resource-poor settings. In endemic regions, epidemics have frequently been reported following heavy rainfalls [2, 13]. Even in industrialized nations including the United States, outbreaks have been reported following sporting events [14–16], within military personnel [17–19], and in tourists [20, 21]. Additionally, there are increasing cases of the disease involving inner-city populations [7, 22], climate changes [23, 24], and expansion of urban slum populations [13, 25]. *L*. *interrogans* are transmitted from contaminated water, soil, or urine to hosts during contact with abraded skin or mucous membrane. Unlike other pathogenic spirochetes, which cause borreliosis or syphilis in humans and are unable to persist outside of a host body, leptospires can persist in aqueous environments for extended periods of time [26, 27]. The pathogen can quickly upregulate genes associated with host adaptation and virulence and can establish serious systemic infection via hematogenous dissemination to multiple internal organs, particularly the kidneys and liver [5, 28, 29]. While wild rodents serve as major natural reservoir hosts, humans and many other domesticated animals are accidental hosts in the transmission cycle of leptospirosis [1, 3, 28].

Pathogenic *Leptospira spp* cause a spectrum of clinical symptoms ranging from mild febrile disease to severe manifestations such as Weil’s disease and pulmonary hemorrhage syndrome, with case fatalities of >10% and >50%, respectively [1, 3, 5, 30]. Although whole cell and recombinant vaccines are shown to interfere with *Leptospira* infection [31–33], none of these vaccines offer complete protection. Moreover, they fail to block chronic renal colonization or urinary shedding, can elicit moderate side effects and are mostly effective against local host-adapted serovars [32, 34, 35]. Thus, given an absence of effective vaccines, prevention of disease progression is primarily reliant on timely diagnosis and antibiotic treatment. Early diagnosis of leptospirosis generally leads to effective antibiotic treatment, thereby preventing the more severe form of disseminated disease; however, there is a lack of rapid diagnostics [8]. Due to the non-specific clinical manifestations of leptospirosis, failure to diagnose the infection, or misdiagnosis, has become a significant problem in many developing countries where dengue, malaria, typhoid and other causes of acute fever are endemic [5, 36]. Diagnosis of leptospirosis still relies on classical laboratory tests including immunoassays against spirochetes or recombinant proteins, direct cultivation of bacteria grown from body fluids, or a microscopic agglutination test (MAT) using paired serum samples and *Leptospira* cultures [8, 37]. Although some of the immunoassays are highly sensitive, they suffer from inherent delays and variability of host immune responses as well as sequence divergence in target antigens, potentially limiting their use for early diagnosis of leptospirosis. Even the gold standard methods of direct culture and the MAT, require either weeks to grow spirochetes from body fluids or highly trained laboratory personnel and paired sera. Therefore, there is a critical need for rapid and effective diagnostics, especially for detection of early infection.

Leptospires disseminate hematogenously and spirochetemia is detectable for many days following initial exposure [3, 8, 38]. Although PCR-based diagnostic methods have been developed that can detect leptospiral DNA [8, 39–50], overall sensitivity of these assays is poor, and in general is less than 60% [8], although in some cases, higher sensitivities are reported [44, 45]. Unlike DNA targets, which usually exist as a single copy per cell, each bacterium contains hundreds to thousands of specific RNA molecules. We therefore hypothesized that an assay based on the PCR amplification of cDNA molecules representing highly and consistently transcribed *Leptospira* genes like16S rRNA [6, 51], which are also mostly conserved in pathogenic *Leptospira* [6, 52], could improve the sensitivity of *Leptospira* detection. In addition, detection of *Leptospira* transcripts in the blood would facilitate prompt and appropriate antibiotic treatment. In the current study, we report a rapid, sensitive, and specific RNA-based PCR diagnostic test for early human leptospirosis. These results could serve as a paradigm for development of novel RNA-based diagnostics of additional bacterial infections in humans, such as Lyme disease, where early diagnostics remains challenging.

## Material and Methods

### Ethics statement

Written informed consent was obtained from all participants prior to blood collection. The study protocol was approved prior to study initiation by the Yale Institutional Review Board (HIC#1006006956), the Ethics Committees at the Oswaldo Cruz Foundation (505.490; 16/2013) and Hospital Couto Maia (175), and the Brazilian Ministry of Health National Ethics Committee in Research (15925). Animals were treated in compliance with the Guide for the Care and Use of Laboratory Animals. All experiments involving human blood, infectious agents, and animals were performed according to the guidelines of the Institutional Biosafety Committee of the University of Maryland, and the Institutional Animal Care and Use Committee of the University of Maryland under the protocol number R-13-71.

### Bacterial strains

*Leptospira* strains and serovars used in the study are indicated in S1 Table. Unless stated otherwise, *Leptospira interrogans* Fiocruz L1-130, a clinical isolate, [53] was used in most parts of the study. In some experiments, additional *Leptospira* serovars were also used, including isolates from 17 pathogenic and five non-pathogenic strains [54]. Spirochetes were grown in liquid Elinghausen-McCullough-Johnson-Harris (EMJH) medium [55, 56] at 29°C on a rotating platform at 100 rpm. Additional bacterial strains, such as an *Escherichia coli* K-12 derivative, Group A *Streptococcus* D471 cells [57], and *Borrelia burgdorferi* clone B31-A3 [58], were also used in certain experiments and grown by using the standard media and protocols.

### Samples

Blood samples from uninfected Golden Syrian Hamsters (4–6 weeks old, purchased from Charles River Laboratories), a species commonly used as an animal model of Leptospirosis [29], were collected into BD Vacutainer whole blood collection tubes containing EDTA (BD Diagnostics). For human samples, twenty five patients were enrolled during active surveillance for leptospirosis at a state-run hospital, Hospital Couto Maia, in Salvador, Brazil, from June 2013 to September 2013, and blood samples were collected during early hospitalization [2]. All patients were examined for leptospirosis using previously described methods (hemoculture and MAT) [30] as well as DNA qPCR. Confirmed patients were positive for at least one of the above three tests. We determined that 22 out of 25 subjects had laboratory-confirmed leptospirosis as defined by: 1) four-fold increase in MAT titer or seroconversion (0 to ≥1:200) between paired sera [59], 2) reciprocal MAT titer of greater than 1:800 in one or more samples [59], 3) positive hemoculture, or 4) positive blood DNA PCR results. Three patients had probable leptospirosis based on the presence of a single MAT titer value of 1:100–1:400.

For samples used in the current study, about 1–2 mL of venous blood was collected directly into BD whole blood EDTA tubes. Within 5 hours of collection, 250 μL of patient blood was aliquoted from the EDTA tubes into 750 μL of TRIzol LS, thoroughly homogenized, and immediately frozen at -70°C. All patient samples were bar-coded, monitored during transport for temperature, and all cold chain data including sample receipt, processing time, and freezing time were recorded. Whole blood from 24 healthy individuals residing in non-endemic regions in the U.S. and Brazil were also collected or purchased (SeraCare Life Sciences) and processed equivalently to patient samples.

### Extraction of nucleic acids and cDNA synthesis

Total RNA samples were extracted from samples stored in TRIzol (for bacterial pellets harvested from cultures grown at mid to late log phases), or TRIzol-LS (for blood samples) according to manufacturer’s (Life Technologies) instructions [60, 61]. After phase separation, RNA samples were either precipitated with isopropanol, dissolved in 20μL of RNase-free water and subjected to optional DNase1 treatment (NEB laboratories), or further purified using an RNeasy mini kit (Qiagen). For cDNA synthesis, 0.5 μg of RNA samples was reversed transcribed using VILO superscript master mix using random primers (Life technologies) according to manufacturer’s protocols. For extraction of DNA, spiked blood samples were processed using DNeasy mini kit (Qiagen) according to manufacturer’s instructions and eluted into 100 μL nuclease-free sterile water.

### Primer design

The primers used for qPCR reaction were designed using NCBI Primer-BLAST primer design program based on the available *L*. *interrogans* genomic sequences. To identify primers of greatest sensitivity and specificity, we aligned the 16S sequences of 37 *Leptospira* serovars, including all 20 known pathogenic or non-pathogenic leptospiral species, and several other non-target bacterial species using MegAlign program (DNASTAR) (**S1 Fig**). We have designed specific sets of 16S forward and reverse primers expected to amplify a specific region of 16S gene that retains 1) absolute homology between all known highly pathogenic *Leptospira* species and serovars, 2) few base pair mismatches for intermediate pathogenic leptospires and 3) greater number of mismatches to non-pathogenic *Leptospira* species. The specificity of each newly designed primer was initially searched against all reference mRNA sequences using NCBI BLASTn as well as Primer-BLAST programs to rule out possible cross-reactivity with other bacteria species as well as non-targeted species including human, mice, rats, and hamsters. These primer sequences display 25% or more divergence from corresponding human or rodent genes, including at least 4–5 base pair mismatches predominantly towards the 3’ ends of the primer sequence inhibiting primer annealing to unintended targets. Similarly, primers against additional gene targets, FlaB, LipL31, LipL32, and LipL41 were also designed. All PCR primer pairs had a similar annealing temperature (60°C) and spanned nearly 200 base pairs of each of the target genes. Prior to their use in RNA measurement assays, each primer pair was tested for efficiency and non-specific amplification by melt-curve analysis using *L*. *interrogans* genomic DNA as a template.

### Polymerase chain reaction

The oligonucleotide sequences for each of the primers used in specific PCR reactions are indicated in S2 Table. The relative levels of cDNA templates in each sample were assessed by quantitative PCR (qPCR) as detailed [60, 61], and whenever necessary, DNA contamination in each sample was measured using an equal volume of purified RNA as a template. All qPCR reactions were performed using the CFX96 real time PCR detection system (Bio-Rad) with the following thermal cycle conditions: 95°C for 10 min, 45 cycles of [95°C for 15 s, 60°C for 60 s], followed by a melt curve from 65°C to 95°C performed at an increment of 0.5°C per cycle. All qPCR plates included no template control wells to test for non-specific amplification or reagent contamination, and results were further tested for specificity by melt curve analysis. Detection of human or hamster *β-actin* or GAPDH transcripts by qPCR confirmed the integrity of cDNA samples. For detection of leptospirosis in humans, an optimized DNA qPCR analysis was also performed using nuclease (TaqMan) assay and primers that amplified a sequence of *lipL32*, a pathogenic Leptospira-specific gene, as detailed earlier [62, 63].

### Validation of primers

*L*. *interrogans* culture was harvested at 2.9x10^8^ leptospires per ml by centrifugation at 5000 g at room temperature (~2.9x10^9^ total cells) and subjected to RNA isolation as detailed above. For relative assessment of each primer set, 2.5 μg RNA samples were reverse transcribed into cDNA, serially diluted to tenfold, which were used in the qPCR reactions. Standard curves and amplification efficiency of the reactions were calculated by the CFX96 instrument software, as instructed by the manufacturer. cDNA samples were also isolated from a number of pathogenic, intermediate pathogenic or non-pathogenic species and serovars. For spiking experiments, 250 μL of untreated whole human blood samples were spiked in triplicate with *L*. *interrogans* derived from at least three independent cultures at various concentrations ranging from 10^6^–10^0^ cells/mL. Samples were used for DNA or RNA extraction using DNeasy mini kit or TRIzol extraction procedure, followed by cleanup using RNeasy mini kit, respectively, and the RNA samples were further processed for cDNA synthesis. As controls for assessment of specificity, cDNA samples were also isolated from additional bacterial culture grown at late log phases including *B*. *burgdorferi*, *E*. *coli*, and Group A *Streptococcus* strains as well as from human blood and hamster liver.

### RNA stability studies

Triplicate samples of 250 μl whole human blood were spiked with viable *L*. *interrogans* (100 cells/ml) derived from at least three independent cultures. Aliquots were homogenized with 750 μLTRIzol LS and stored at room temperature for 0, 4, 8, 24, 72, or 120 h before freezing at -80°C until analysis. In parallel experiments, triplicate aliquots of 250 μL human blood containing 100 leptospires per ml were stored at room temperature, 4°C, -20°C, or -80°C in the absence of any stabilization reagent, and processed together with other conditions. Samples were collected directly into 750 μL TRIzol LS after incubating at the aforementioned temperatures for 0 h, 8 h, 24 h, 7 d, and 14 d, and stored at -80°C until completion of all timepoints. RNA was extracted using the TRIzol procedure, further treated with DNase I, and finally reverse transcribed to cDNA and analyzed by qPCR using *Leptospira*-specific 16S primers.

### Statistical analysis

Results are expressed as the mean ± standard error of the mean (SEM). The significance of the difference between the mean values of the groups was evaluated by unpaired Student *t* test and ANOVA.

## Results

### Development of an RNA-based quantitative PCR assay for detection of pathogenic leptospires

**Fig 1:**
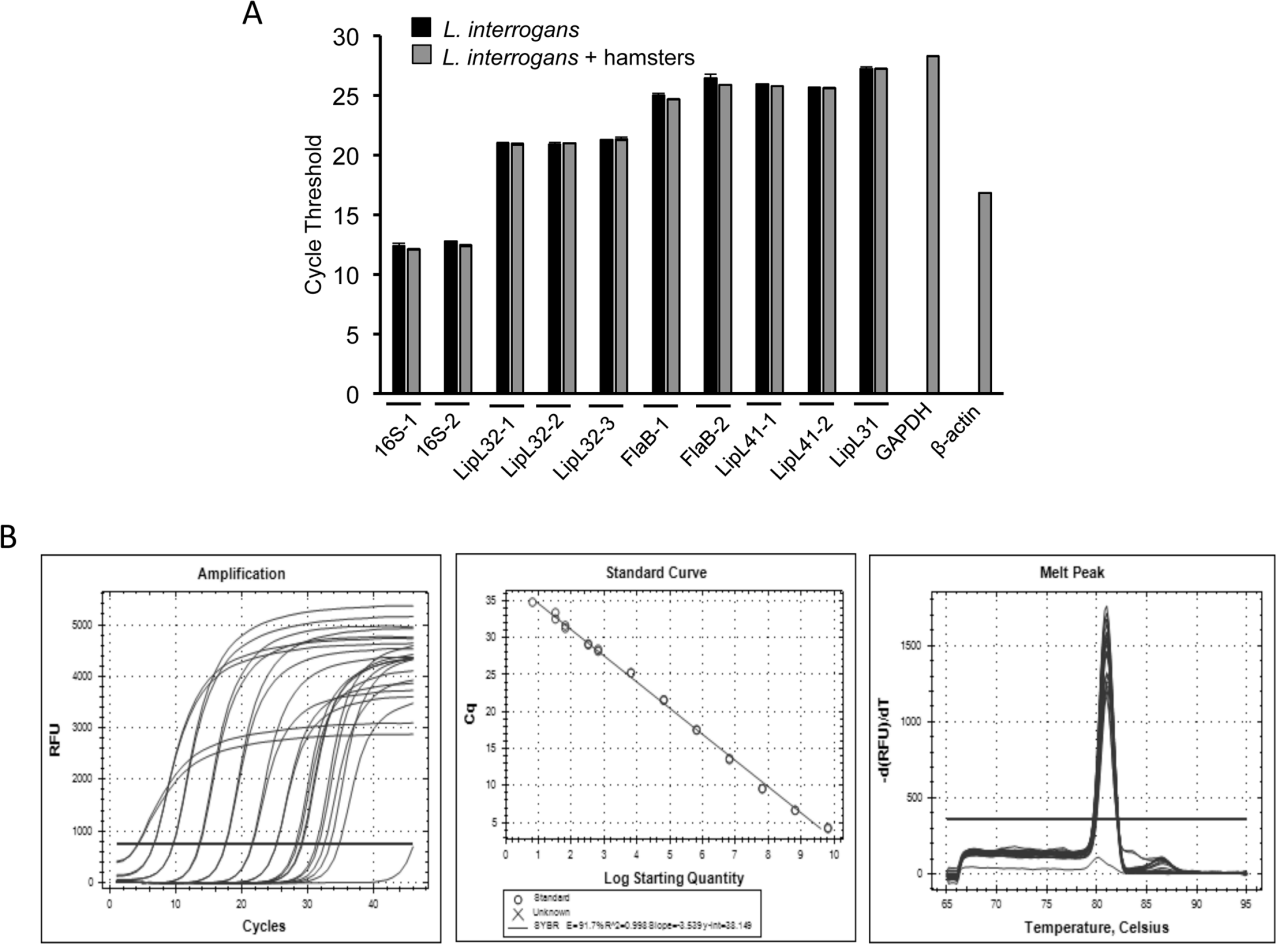
*L*. *interrogans* 16S rRNA primers offered highest analytical sensitivity. A) Total RNA samples isolated from cultured spirochetes were converted into cDNA and amplification cycles (cycle threshold or Ct value) of various *L*. *interrogans* target genes are assessed in qRT-PCR assays in the presence (gray bars) or absence (black bars) of hamster cDNA. Data represent results from three independent experiments. B) 16S rRNA primers display a high PCR efficiency. *L*. *interrogans* cDNA in RNase-free water (320 ng/μL) was serially diluted to tenfold (10^−1^ to 10^−9^) and subjected to qRT-PCR assays using 16S-1 primers. Amplification cycles (left panel) were used to calculate standard curve (middle panel), which indicated detection to 10^−9^ dilutions with an amplification efficiency of 91.2%. A melt curve analysis (right panel) showed a melting temperature of 82°C without any non-specific amplification.

Development of a rapid and sensitive diagnostics for human leptospirosis, especially for detection of early active infection, is highly warranted. Since pathogenic leptospires are known to disseminate via blood where they are detectable for several days after infection [8], and since each bacterium may contain hundreds of copies of certain abundant transcripts, we sought to explore whether an RNA-based PCR assay would allow sensitive and specific detection of leptospires in human blood. We adopted a SYBR Green-based qRT-PCR assay, which is a highly efficient and widely used platform amongst available real-time PCR technologies [64], yet relatively simple and cost-effective. For identification of an RNA target that yields most efficient detection, we initially examined a set of characterized, abundant rRNA and mRNA gene targets: 16S rRNA, FlaB, LipL31, LipL32, and LipL41. We selected these genes not only due to their constitutive and abundant expression but also for their sequence conservation in pathogenic and intermediately pathogenic *Leptospira spp*, and sequence divergence or absence in non-target species. Although the above-mentioned mRNA genes are unique to pathogenic *Leptospira* species [6, 52], specific regions of their rRNA genes display appreciable species-specific conservation [65]. We therefore used NCBI Primer-Blast software to identify unique regions in 16S gene and created forward and reverse primers 100% identical to pathogenic *L*. *interrogans* sequences but containing several nucleotide mismatches to non-target bacterial species including non-pathogenic *Leptospira* (**S1 Fig**). These primers also lack significant similarity to mammalian species. All gene-specific primers had a similar annealing temperature and comparable amplicon sizes. The RNA samples from a highly pathogenic species, *L*. *interrogans* serovar Copenhageni strain Fiocruz L1-130 were converted into cDNA and used in a SYBR Green-based qPCR assay in the absence or presence of 10-fold excess of a control host (hamster) cDNA. While sensitivity of the assay (or the relative abundance of the transcripts) was calculated using the 2^−ΔΔCT^ method [66], specificity of the target gene amplification was assessed using melt-curve analysis. We found that the 16S rRNA primers offered the most efficient analytical sensitivity, as evidenced by the lowest Ct values, and were nearly 1000-fold more abundant than the next efficient mRNA target, LipL32 (**Fig 1A**). The 16S-1 primers (**Fig 1B**), or other tested primers, showed PCR efficiencies between 91–99% and without detectable cross-reactivity with spiked control rodent cDNAs. As the 16S rRNA primers offered the highest sensitivity in our assay, we only used these primers in subsequent experiments (16S-1, Fig 1).

### Analytical sensitivities of 16S RNA-based qPCR assays are superior to corresponding DNA-based assay in *Leptospira*-spiked human samples

**Fig 2:**
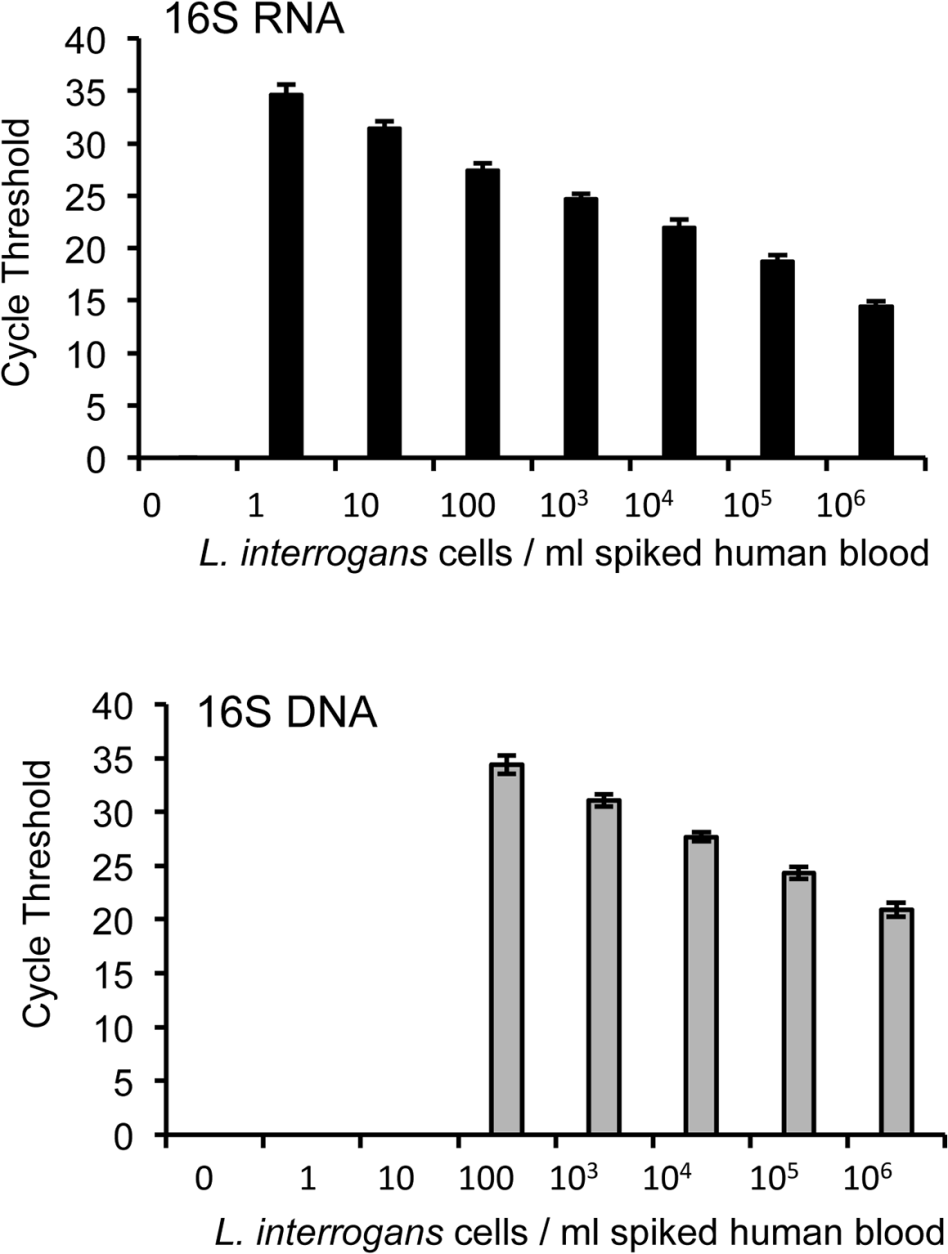
RNA-based detection is more sensitive than DNA. Aliquots of *L*. *interrogans* bacteria were serially diluted from 10^6^ to 1 bacterium per milliliter of human blood and used for either RNA-based qRT-PCR (upper panel) or DNA-based qPCR (lower panel). Data represent results from three independent experiments.

We next assessed whether sensitivity of our 16S RNA-based assay is superior to corresponding DNA-based ones and also tested its specificity with experimentally spiked human blood samples. To accomplish this, we spiked serially diluted *L*. *interrogans* cells into 250 μl aliquots of human blood and the RNA and DNA samples were isolated using the commercial kits as detailed in the Materials and Methods. We then performed subsequent qRT-PCR and qPCR assays using corresponding templates and the same 16S primer set. Using this methodology, we found that RNA-based assays were at least 100-fold more sensitive than a DNA-based approach (**Fig 2**), suggesting that the difference in assay performance is not due to the target sequence rather than differences between RNA and DNA detection.

### Differential detection of multiple *Leptospira* and non-target bacterial species

**Fig 3:**
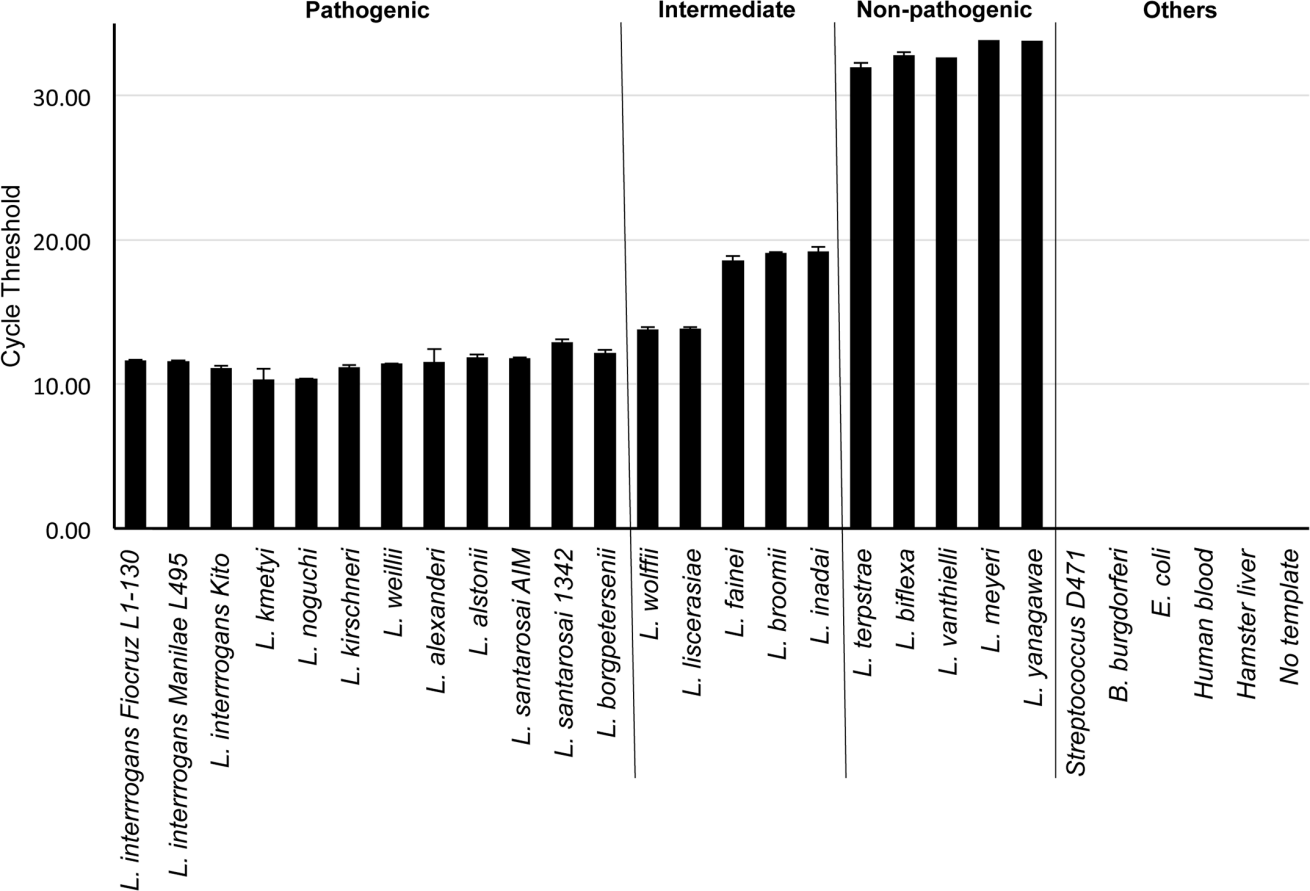
High sensitivity and specificity of 16S rRNA qPCR assay for pathogenic and nonpathogenic leptospiral species and serovars. Total RNA was extracted from 17 high or intermediate pathogenic, and five non-pathogenic leptospiral species, *Borrelia burgdorferi*, group A *Streptococcus*, and *Escherichia coli*, as well as from uninfected human blood or hamster liver, as described in the materials and methods and converted into cDNA. Equal amount (10 ng) of cDNA templates from each bacterial species were subjected to qRT-PCR assays using 16S-1 primers and amplification cycles (Ct values) were measured. Note that the sensitivity of detection is the best for all tested highly pathogenic species or serovars followed by intermediate species while non-pathogenic strains display the lowest sensitivity (an average of 15 Ct values or 10,000 fold less detectability). All tested non-target bacterial species or mammalian samples remained undetectable. Data represent results from three independent experiments.

We next determined the sensitivity of our 16S qRT-PCR assay in the detection of multiple pathogenic leptospiral serovars, and the specificity of the assay using non-pathogenic leptospiral species and non-target bacterial species. For this purpose, we prepared RNA samples from 17 highly or intermediately pathogenic leptospiral species and serovars as well as from five non-pathogenic species, *B*. *burgdorferi*, group A *Streptococcus*, *E*. *coli* or hamster and human tissues. We found that while the assay was able to detect all pathogenic species with highest sensitivity, detection of non-pathogenic leptospiral species was at least 15 Ct value (or 10,000 fold) higher (**Fig 3**). These results indicate a difference of several thousand folds in the concentration of target templates. In addition, weak signals at higher Ct values (39–40) were recorded for *Streptococcus* and *Borrelia*, however, this amplification was non-specific, as confirmed by the melt curve analysis. Thus, overall, we detected none of the non-target species suggesting 100% specificity of the assay (**Fig 3**).

### *L*. *interrogans* 16S transcripts are remarkably stable in human blood

**Fig 4:**
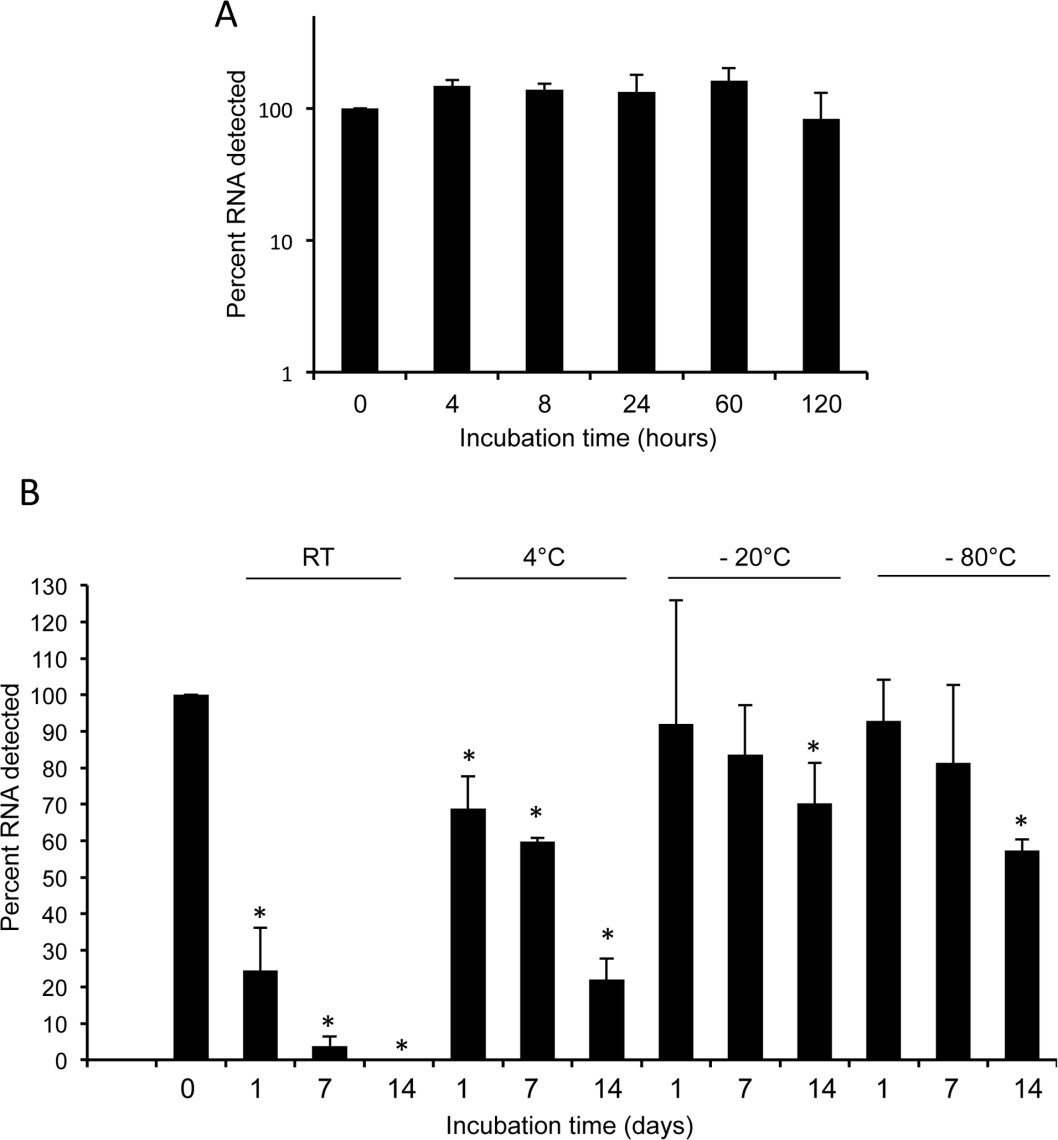
Stability of *Leptospira* 16S transcripts in human blood. A) Transcript stability in the blood treated with an RNA stabilization agent. Aliquots of human blood were spiked with leptospires (100 cells/ml), mixed with an RNase stabilization agent (TRIzol), and each aliquot was stored at room temperature for various times (0–120 hours). Following storage, levels of 16S rRNA transcripts were measured using qRT-PCR assays. Data represent results from three independent experiments. B) Transcript stability in the blood stored at various temperatures in the absence of any RNA stabilization agent. Spiked samples were prepared as described above and stored either at room temperature or at various cold temperatures (4°C, -20°C, and -80°C) up to 14 days, and transcript levels were monitored by qRT-PCR analyses. Transcript levels of “0 hour” were considered as 100%, which served as baseline controls, which displayed significant differences in transcript levels in groups marked by an asterisk (ANOVA, p<0.05). Data represent results from three independent experiments.

An ideal RNA-based diagnostic test for leptospirosis would detect stable targets, allowing for varied blood storage times and temperatures, and not require the use of toxic RNA stabilizing reagents such as TRIzol. Therefore, we compared the limit of detection of 16S transcripts in spiked human blood samples stored at room temperature in TRIzol to that of untreated spiked blood samples. We also tested how storage temperature of blood samples influenced the window of 16S detection by our assay. To accomplish our objectives, we spiked human blood with *L*. *interrogans* cells at 100 bacteria per milliliter of blood and stored aliquots at room temperature, 4°C, -20°C, or -80°C) for 1, 7 and 14 days either with or without the addition of TRIzol. A sample immediately stored in TRIzol and frozen served as the “0 hour” or baseline control for 16S RNA stability experiments. We measured the transcript levels by qRT-PCR analyses, and observed no appreciable RNA degradation in the samples stored in TRIzol at room temperature (**Fig 4A**) or at colder temperatures. Of note, while we recorded a significant loss of 16S RNA in samples stored at room temperature within a day, ~ 5% of the transcripts remained detectable until 7 days (**Fig 4B**). In contrast, we were able to detect >20% of 16S RNA transcripts from samples stored at 4°C, and >50% at -20°C and -80°C even after 14 days (**Fig 4B**).

### Assessment of clinical cases of human leptospirosis

Finally, we tested the sensitivity and specificity of our assay for the detection of *Leptospira* in blood samples obtained from patients, with suspected leptospirosis, as detailed in the Material and Methods section. We also compared the efficacy of our RNA assay using an established DNA-based qPCR assay. Total RNA and DNA samples were isolated from these human samples, and detection of *Leptospira* was examined by real-time PCR analyses, as detailed in the Materials and Methods section. Results indicated that, for 22 confirmed leptospirosis cases, *Leptospira* RNA was detected in 14 samples, yielding a sensitivity of 64% (Table 1). In parallel, an established qPCR assay for detection of leptospiral DNA form the corresponding human samples, as described in the Materials and Methods, identified 7 positives, thereby reflecting a significantly lower sensitivity (32%, p = 0.035) for the DNA detection assay compared to the RNA detection assay. Of note, 2 of the 3 probable clinically suspected cases were found to have detectable leptospiral RNA, whereas none of the probable cases had detectable DNA. As expected, all blood samples obtained from the 24 control individuals failed to yield a positive signal, suggesting a 100% specificity of our RNA-based assay.

**Table 1:** Detection of leptospiral RNA by quantitative PCR in blood samples collected from humans with suspected leptospirosis.

Assay results	Confirmed leptospirosis (N = 22)	Probable leptospirosis (N = 3)	Healthy Subjects (N = 24)
	Number (%)		
RNA and DNA PCR+	5 (23)	0 (0)	0 (0)
RNA PCR+ alone	9 (41)	2 (67)	0 (0)
DNA PCR+ alone	2 (9)	0 (0)	0 (0)
Total RNA PCR+	14 (64)	2 (67)	0 (0)
Total DNA PCR+	7 (32)	0 (0)	0 (0)

## Discussion

As leptospirosis affects nearly a million people annually [8] and an efficient human vaccine is unavailable, development of a better diagnosis test is critical for effectively treating patients with leptospirosis. However, efficient detection of the infection is difficult to accomplish, primarily due to the fact that pathogenic leptospires not only share features of both gram-positive and gram-negative bacteria including other related spirochetes, clinical manifestation of the infection also share features of many prevalent undifferentiated febrile illnesses, such as influenza, dengue and malaria [37, 67–69]. Here, we report a use of leptospiral RNA as a diagnostic target for development of a rapid, sensitive and specific quantitative PCR assay for detection of human leptospirosis. While RNA-based diagnostic methods for detection of bacterial pathogens are uncommon, except for a few commercially-available assays for detection of certain sexually transmitted bacterial infections [70–72], to the best of our knowledge, this is the first example of an RNA-based diagnosis of human leptospirosis. Our discovery of the relative stability of 16S transcripts in untreated stored human blood samples indicate that the RNA-based assay could be widely applied for the diagnosis of leptospirosis. Additionally, our results suggest similar approaches can be employed to develop novel diagnostic tests for other bacterial diseases.

Several serological immunoassays are available to date for detection of leptospirosis [8, 37], however, diagnosis of human or animal infection is still inadequate, which is based on classical microbiological methods. The gold standard method for such diagnosis is culture or microscopic agglutination test (MAT), which are extremely slow procedures that also suffer from sensitivity, especially for diagnosis of early infection. Newer diagnostic methods that allow diagnosis of early infection at a relatively fast pace and facilitate initiation of prompt antibiotic treatment could alleviate more severe complications of disseminated infection. As spirochetemia is likely to be associated with early infection, which occurs for at least two weeks following initial infection, we hypothesized that the detection of nucleic acids with sequence specificity to pathogenic *Leptospira* could surrogate an active infection. Such detection in turn would yield high diagnostic efficiency if the transcripts or corresponding RNA fragments are abundant, and remain stable, in stored blood. Unlike conventional genomic DNA molecules that mostly represent a single copy per bacterial cell, or mRNA molecules, which constitute the minor population of total cellular RNA, ribosomal RNA molecules are likely to be abundant and thus offer more promising diagnostic target. In particular 16S rRNA, due to its high expression, and maintenance of species-specific sequences [65], has been widely used for taxonomic studies or as a diagnostic target to identify a particular bacterial species. Interestingly, unlike other major pathogenic spirochetes like *Borrelia burgdorferi* [73], *L*. *interrogans* genome houses at least two copies of 16S rRNA genes also conserved in sequenced genomes of pathogenic leptospires [6, 52]. Thus, it is perhaps not surprising that compared to even abundantly and consistently-expressed mRNA gene targets like *lipL32* or *flaB* [51], our assay targeting 16S rRNA achieves better sensitivity that allow detection of low numbers of *L*. *interrogans* cell per milliliter of spiked human blood. This is also a notable improvement in sensitivity, compared to existing DNA-based PCRs where the limit of detection ranges from 10^2^–10^3^ bacteria per milliliter of blood or urine [8, 40–42]. Leptospiral 16S RNA molecules remain appreciably stable in the blood, further highlighting their potential use in the diagnosis of early infection. We do not know how 16S RNA molecules maintain notable stability in blood, however, this could be contributed by the remarkable spirochete ability to remain viable in certain aqueous environment *ex vivo*. In addition, existence of intact or fragmented 16S transcripts in the blood, or within phagocytic cells that enable their detection, also remains as interesting possibilities.

Although diagnosis of microbial infection based on the detection of a target RNA molecule offers multiple advantages, such as higher sensitivity and specificity, or potential indicator of early and active infection, there are inherent limitations that influence successful development of RNA PCR-based diagnostics. RNA molecules are generally less stable than other biomarkers and their cellular abundance (and thus detection) are likely to be variable, which could also be influenced by a number of additional factors. For example, efficiency of RNA detection depends on successful reverse transcription or absence of intrinsic blood factors that inhibit reverse transcription or subsequent PCR. In addition, transcript abundance may vary from bacterial cell to population levels, growth stages, or environment. Thus, absolute quantitation of microbial cells based on enumeration of RNA molecules might not be possible. Despite these challenges, due to their outstanding abundance and specificity, as also highlighted in our study, RNA targets are used in the diagnosis of a number of human infections, primarily the ones caused by viruses [74–77] and in a limited number of cases, for detection of bacterial infections, such as *Mycobacteria* species [78, 79], or *Chlamydia* trachomatis and *Neisseria gonorrhoeae* [70–72]. To the best of our knowledge, the current study represents the first attempt to use RNA detection to develop an improved diagnostic test for leptospirosis. Notably, 16S gene sequences were recently targeted for development of a DNA based real-time PCR assay, denoted as rt-PCR [50], which used additional primers tagged with reporter dye and quencher molecules called TaqMan probes to enhance analytical specificity, however the assay achieved overall clinical sensitivity of 34%. While our RNA assay yielded relatively superior sensitivity, we also show that use of a simpler in-house platform like SYBR Green-based qPCR assay could retain comparable specificities of TaqMan-probe based PCR. Nevertheless, we recognize that our results need to be further validated for true sensitivity and specificity values using additional studies involving larger numbers of patients, including ones from diverse epidemiological settings beyond hospitalized patients from Salvador, Brazil involved in the current study.

Although currently overall sensitivity of our RNA assay is twice as high as parallel TaqMan probe-based DNA assays and yielded statistically significant differences, it detected leptospiral RNA in 64% of the laboratory-confirmed cases. The exact reasons why our assay failed to yield positivity in a subset of laboratory-confirmed samples, including two patients where DNA assays are also positive, remained puzzling, however, could be linked to the effectiveness of antimicrobial therapy influencing spirochetemia at the time of individual sample collection, or unintended degradation of 16S RNA during less careful handling and storage conditions, amongst other unknown possibilities. On the other hand, notably, our RNA assay detected positivity in two human samples where routine laboratory diagnosis remained unconfirmed. While this could be interpreted as potential loss of specificity, it also suggests enhanced sensitivity of the RNA assay, especially in cases where pathogens are rapidly cleared before development of detectable humoral immune responses. The latter speculation of greater sensitivity of our assay is supported by its high (100%) specificity, where samples from normal humans failed to yield positivity. Notably, an earlier study also reported positivity in clinical diagnosis of *Leptospira* by PCR and sequencing methods in a subset of clinical samples that are serologically negative using a standard MAT panel [39]. Nevertheless, while implementation of additional care towards sample collection and storage would further enhance sensitivity of our RNA assay, use of a real-time PCR platform already has advantage over current serology-based assays in terms of rapidity, allowing quicker diagnosis of early infection and prompt antimicrobial treatment, which could prevent more severe and life-threatening complication of disseminated infection. In addition to currently-adopted SYBR Green-based qPCR assay, our RNA detection strategy is amenable to additional cheaper nucleic acid amplification methods, such as Loop Mediated Isothermal Amplification (LAMP) reaction for detection of a variety of human pathogens [80–87], including leptospirosis [88]. We also envision that future development of more efficient 16S primers or PCR platforms as well as combinatorial use of multiple gene targets could further improve the sensitivity and specificity of our assay. Therefore, our study could have far-reaching implications for development of simple, cost-effective, and rapid RNA-based PCR assays for detection of human leptospirosis as well as other bacteremic human pathogens where efficient diagnosis of early or active infection is warranted yet remains as an unmet need.

## Supporting Information

S1 Fig Alignment of 16S rRNA gene sequences from known leptospiral species and serovars and other non-target bacterial species targeted by the primers. Nucleotide sequences within the primer sequences of 16S rRNA genes from 37 *Leptospira* serovars, including all 20 known species, as well as non-target bacteria including other major pathogenic spirochetes, were derived from NCBI nucleotide sequence databases and aligned using MegAlign (DNASTAR software). Base pair mismatches between *L*. *interrogans* Fiocruz L1-130 and other species are indicated by boxes.
(PDF)

S1 Table Leptospira species and serovars used in the study. (PDF)

S2 Table Oligonucleotide primers used in the study. (PDF)
