# Supplementary material for: Efficient Detection of Pathogenic Leptospires Using 16S Ribosomal RNA
Source: PLoS One. 2015 Jun 19;10(6):e0128913. doi: 10.1371/journal.pone.0128913 (PMC4474562; doi:10.1371/journal.pone.0128913)
Supplement: S1 Fig — Nucleotide sequences within the primer sequences of 16S rRNA genes from 37 Leptospira serovars, including all 20 known species, as well as non-target bacteria including other major pathogenic spirochetes, were derived from NCBI nucleotide sequence databases and aligned using MegAlign (DNASTAR software). Base pair mismatches between L. interrogans Fiocruz L1-130 and other species are indicated by boxes. (PDF) [file pone.0128913.s001.pdf]

S1 Fig.

16S-1 forward

Majority	GCGTAGGCGGACATGTAAGT
	10 20
Leptospira interrogans serovar Copenhag	GCGTAGGCGGACATGTAAGT
Leptospira interrogans serovar Icteroha	GCGTAGGCGGACATGTAAGT
Leptospira interrogans serovar Icteroha	GCGTAGGCGGACATGTAAGT
Leptospira interrogans serovar Icteroha	GCGTAGGCGGACATGTAAGT
Leptospira interrogans serovar Lai str.	GCGTAGGCGGACATGTAAGT
Leptospira interrogans strain RGA.seq	GCGTAGGCGGACATGTAAGT
Leptospira kirschneri serovar Cynopteri	GCGTAGGCGGACATGTAAGT
Leptospira noguchii serovar Panama stra	GCGTAGGCGGACATGTAAGT
Leptospira noguchii serovar Panama stra	GCGTAGGCGGACATGTAAGT
Leptospira noguchii serovar Panama stra	GCGTAGGCGGACATGTAAGT
Leptospira alexanderi serovar Manhao 3	GCGTAGGCGGACATGTAAGT
Leptospira weilii serovar Celledoni str	GCGTAGGCGGACATGTAAGT
Leptospira weilii strain Celledoni.seq	GCGTAGGCGGACATGTAAGT
Leptospira borgpetersenii serovar Javan	GCGTAGGCGGACATGTAAGT
Leptospira borgpetersenii serovar Javan	GCGTAGGCGGACATGTAAGT
Leptospira borgpetersenii serovar Hardj	GCGTAGGCGGACATGTAAGT
Leptospira santarosai serovar Shermani	GCGTAGGCGGACATGTAAGT
Leptospira santarosai serovar Shermani	GCGTAGGCGGACATGTAAGT
Leptospira kmetyi serovar Malaysia stra	GCGTAGGCGGACATGTAAGT
Leptospira broomii strain 5399.seq	GCGTAGGCGGATTTGTAAGT
Leptospira fainei serovar Hurstbridge s	GCGTAGGCGGATTTGTAAGT
Leptospira fainei serovar Hurstbridge s	GCGTAGGCGGATTTGTAAGT
Leptospira inadai serovar Lyme strain 1	GCGTAGGCGGATTTGTAAGT
Leptospira licerasiae serovar Varillal	GCGTAGGCGGACATGTAAGT
Leptospira wolffii serovar Khorat strai	GCGTAGGCGGACTTGTAAGT
Leptospira biflexa serovar Patoc strain	GTGTAGGCGGACTAACAAGT
Leptospira biflexa serovar Patoc strain	GTGTAGGCGGACTAACAAGT
Leptospira biflexa serovar Patoc strain	GTGTAGGCGGACTAACAAGT
Leptospira meyeri serovar Ranarum strai	GTGTAGGCGGACTAACAAGT
Leptospira wolbachii serovar Codice str	GTGTAGGCGGACTAACAAGT
Leptospira wolbachii strain CDC BIFLEXA	GTGTAGGCGGACTAACAAGT
Leptospira vanthielii serovar Holland s	GTGTAGGCGGACTAACAAGT
Leptospira terpstrae serovar Hualin str	GTGTAGGCGGACTAACAAGT
Leptospira yanagawae serovar Saopaulo s	GTGTAGGCGGACTAACAAGT
Leptospira idonii 16S ribosomal.seq	GTGTAGGCGGATTAACAAGT
Borrelia sp. B31.seq	GAGTAGGCGGATATATAAGT
Brevibacillus brevis NBRC 100599 47.seq	GCGCAGGCGGCTATGTAAGT
Turneriella parva DSM 21527.seq	TCGCAGGTGGTTTGTTAAGT
Staphylococcus haemolyticus JCSC1435 st	GCGTAGGCGGTTTTTTAAGT
Staphylococcus epidermidis RP62A strain	GCGTAGGCGGTTTTTTAAGT
Staphylococcus aureus subsp. aureus N31	GCGTAGGCGGTTTTTTAAGT
Streptococcus suis BM407 strain BM407.s	GCGCAGGCGGTTTGATAAGT
Streptococcus mutans strain ATCC 25175.	GCGCAGGCGGTCAGGAAAGT
Clostridium difficile 630 strain 630.se	GCGTAGGCGGTCTTTCAAGT
Salmonella enterica subsp. enterica ser	ACGCAGGCGGTCTGTCAAGT
Mycobacterium tuberculosis strain H37Rv	TCGTAGGTGGTTTGTCGCGT
Escherichia coli O157.H7 str. Sakai str	ACGCAGGCGGTTTGTTAAGT
Escherichia coli str. K-12 substr. MG16	ACGCAGGCGGTTTGTTAAGT
Klebsiella pneumoniae subsp. pneumoniae	ACGCAGGCGGTCTGTCAAGT
Borrelia recurrentis A1 strain A1.seq	GAGTAGGCGGATATGCAAGT
Borrelia miyamotoi.seq	GAGTAGGCGGATATGCAAGT
Borrelia hermsii DAH strain DAH.seq	GAGTAGGCGGATATGCAAGT
Borrelia afzelii PKo strain PKo.seq	GAGTAGGCGGATATATAAGT
Borrelia burgdorferi N40 strain N40.seq	GAGTAGGCGGATATATAAGT
Borrelia garinii PBi strain PBi.seq	GAGTAGGCGGATATATAAGT
Borrelia bissettii strain DN127.seq	GAGTAGGCGGATATATAAGT
Treponema pallidum subsp. pallidum SS14	ATGCAGGCGGACTGGTAAGC
Treponema denticola strain ATCC 35405.s	ATGTAGGCGGTTAGGTAAGC

16S-1 reverse

Majority	CGTGGGTAGTGAACGGGATT
	10 20
Leptospira interrogans serovar Copenhag	CGTGGGTAGTGAACGGGATT
Leptospira interrogans serovar Icteroha	CGTGGGTAGTGAACGGGATT
Leptospira interrogans serovar Icteroha	CGTGGGTAGTGAACGGGATT
Leptospira interrogans serovar Icteroha	CGTGGGTAGTGAACGGGATT
Leptospira interrogans serovar Lai str.	CGTGGGTAGTGAACGGGATT
Leptospira interrogans strain RGA.seq	CGTGGGTAGTGAACGGGATT
Leptospira kirschneri serovar Cynopteri	CGTGGGTAGTGAACGGGATT
Leptospira noguchii serovar Panama stra	CGTGGGTAGTGAACGGGATT
Leptospira noguchii serovar Panama stra	CGTGGGTAGTGAACGGGATT
Leptospira noguchii serovar Panama stra	CGTGGGTAGTGAACGGGATT
Leptospira alexanderi serovar Manhao 3	CGTGGGTAGTGAACGGGATT
Leptospira weilii serovar Celledoni str	CGTGGGTAGTGAACGGGATT
Leptospira weilii strain Celledoni.seq	CGTGGGTAGTGAACGGGATT
Leptospira borgpetersenii serovar Javan	CGTGGGTAGTGAACGGGATT
Leptospira borgpetersenii serovar Javan	CGTGGGTAGTGAACGGGATT
Leptospira borgpetersenii serovar Hardj	CGTGGGTAGTGAACGGGATT
Leptospira santarosai serovar Shermani	CGTGGGTAGTGAACGGGATT
Leptospira santarosai serovar Shermani	CGGGGGTAGTGAACGGGATT
Leptospira kmetyi serovar Malaysia stra	CGTGGGTAGTGAACGGGATT
Leptospira broomii strain 5399.seq	CGTGGGTAGTAAACGGGATT
Leptospira fainei serovar Hurstbridge s	CGTGGGTAGTAAACGGGATT
Leptospira fainei serovar Hurstbridge s	CGTGGGTAGTAAACGGGATT
Leptospira inadai serovar Lyme strain 1	CGTGGGTAGTAAACGGGATT
Leptospira licerasiae serovar Varillal	CGTGGGTAGTAAACGGGATT
Leptospira wolffii serovar Khorat strai	CGTGGGTAGTAAACGGGATT
Leptospira biflexa serovar Patoc strain	CGTGGGTAGTGAACGGGATT
Leptospira biflexa serovar Patoc strain	CGTGGGTAGTGAACGGGATT
Leptospira biflexa serovar Patoc strain	CGTGGGTAGTGAACGGGATT
Leptospira meyeri serovar Ranarum strai	CGTGGGTAGTGAACGGGATT
Leptospira wolbachii serovar Codice str	CGTGGGTAGTGAACGGGATT
Leptospira wolbachii strain CDC BIFLEXA	CGTGGGTAGTGAACGGGATT
Leptospira vanthielii serovar Holland s	CGTGGGTAGTGAACGGGATT
Leptospira terpstrae serovar Hualin str	CGTGGGTAGTGAACGGGATT
Leptospira yanagawae serovar Saopaulo s	CGTGGGTAGTGAACGGGATT
Leptospira idonii 16S ribosomal.seq	CGTGGGTAGTGAACGGGATT
Borrelia sp. B31.seq	CGTAGGGAGCAAACAGGATT
Brevibacillus brevis NBRC 100599 47.seq	CGTGGGGAGCAAACAGGATT
Turneriella parva DSM 21527.seq	TGCGGGGAGCAAACAGGATT
Staphylococcus haemolyticus JCSC1435 st	CGTGGGGATCAAACAGGATT
Staphylococcus epidermidis RP62A strain	CGTGGGGATCAAACAGGATT
Staphylococcus aureus subsp. aureus N31	CGTGGGGATCAAACAGGATT
Streptococcus suis BM407 strain BM407.s	CGTGGGGAGCGAACAGGATT
Streptococcus mutans strain ATCC 25175.	CGTGGGTAGCGAACAGGATT
Clostridium difficile 630 strain 630.se	CGTGGGGAGCAAACAGGATT
Salmonella enterica subsp. enterica ser	CGTGGGGAGCAAACAGGATT
Mycobacterium tuberculosis strain H37Rv	CGTGGGGAGCGAACAGGATT
Escherichia coli O157.H7 str. Sakai str	CGTGGGGAGCAAACAGGATT
Escherichia coli str. K-12 substr. MG16	CGTGGGGAGCAAACAGGATT
Klebsiella pneumoniae subsp. pneumoniae	CGTGGGGAGCAAACAGGATT
Borrelia recurrentis A1 strain A1.seq	CGTAGGGAGCAAACAGGATT
Borrelia miyamotoi.seq	CGTAGGGAGCAAACAGGATT
Borrelia hermsii DAH strain DAH.seq	CGTAGGGAGCAAACAGGATT
Borrelia afzelii PKo strain PKo.seq	CGTAGGTAGCAAACAGGATT
Borrelia burgdorferi N40 strain N40.seq	CGTAGGGAGCAAACAGGATT
Borrelia garinii PBi strain PBi.seq	CGTAGGGAGCAAACAGGATT
Borrelia bissettii strain DN127.seq	CGTAGGGAGCAAACAGGATT
Treponema pallidum subsp. pallidum SS14	TGTGGGGAGCGAACAGGATT
Treponema denticola strain ATCC 35405.s	TGCGGGGAGCAAACAGGATT
