# Supplementary material for: Efficient Detection of Pathogenic Leptospires Using 16S Ribosomal RNA
Source: PLoS One. 2015 Jun 19;10(6):e0128913. doi: 10.1371/journal.pone.0128913 (PMC4474562; doi:10.1371/journal.pone.0128913)
Supplement: S1 Table — (PDF) [file pone.0128913.s002.pdf]

**S1 Table: Leptospira species and serovars used in the study**

| Species | Serogroup | Serovar | Strain |
|---|---|---|---|
| **Pathogenic *Leptospira*** | | | |
| *L. alexanderi* | Manhao | Manhoa 3 | L 60T |
| *L. alstoni* | Ranarum | Pingchang | 80-412T |
| *L. interrogans* | Canicola | Canicola | Kito |
| *L. interrogans* | Icterohaemorrhagiae | Copenhageni | Fiocruz L1-130 |
| *L. interrogans* | Pyrogenes | Manilae | L495 |
| *L. borgpetersenii* | Mini | Mini | 200901116 |
| *L. kirschneri* | Grippotyphosa | Grippotyphosa | RM52 |
| *L. kmetyi* | Tarassovi | Malaysia | Bejo-Iso9 |
| *L. noguchii* | Autumnalis | ND | Bonito |
| *L. santarosai* | Shermani | Shermani | 1342 KT |
| *L. santarosai* | Tarassovi | ND | AIM |
| **Intermediate *Leptospira*** | | | |
| *L. broomii* | ND | ND | 5399T |
| *L. fainei* | Hurstbridge | Hurstbridge | BUT 6T |
| *L. inadai* | ND | Lyme | 10T |
| *L. licerasiae* | Iquitos | Varillal | VAR 010 |
| *L. wolffii* | ND | ND | Khorat-H2T |
| **Non-Pathogenic *Leptospira*** | | | |
| *L. biflexa* | Semaranga | Patoc | Patoc1 |
| *L. meyeri* | Sejroe | Hardjo | Went 5 |
| *L. terpstrae* | ND | Hualin | LT 11-33T |
| *L. vanthielii* | ND | Holland | Waz Holland |
| *L. yanagawae* | ND | Saopaulo | Sao Paulo |
