# Supplementary material for: Efficient Detection of Pathogenic Leptospires Using 16S Ribosomal RNA
Source: PLoS One. 2015 Jun 19;10(6):e0128913. doi: 10.1371/journal.pone.0128913 (PMC4474562; doi:10.1371/journal.pone.0128913)
Supplement: S2 Table — (PDF) [file pone.0128913.s003.pdf]

S2 Table: Oligonucleotide primers used in the study

Primer	Amplicon (base pair)	Forward	Reverse
16S-1	211	GCGTAGGCGGACATGTAAGT	AATCCCGTTCACTACCCACG
16S-2	235	TAAAGGCTCACCAAGGCGAC	TTAGCCGGTGCTTTAGGCAG
LipL32-1	190	GCCGTAATCGCTGAAATGGG	CTTTGGCGATTTGGTCAGGC
LipL32-2	262	TGGCTATCTCCGTTGCACTC	CCCATTTCAGCGATTACGGC
FlaB-1	192	GCTCGTGCAGGTGGAAGTAT	GCCTTTGAAGTCATCGTGCC
FlaB-2	183	GCTAACGACGTGATCGGTCT	CGAGACAACTTCTTCCGCCA
LipL41-1	184	GTGCAGACGCAATCAACGAA	GCGAAACCTGCCACTTTCAA
LipL41-2	156	CGTAGGTTTGGCTGTTGAAGC	GCGTCTGCACGTTTACTCAG
LipL-31	180	TCGATGCGATGAGTCGAGTT	AACCGTCTTTTTCAGCTGCG
